# Smoking behaviour, tobacco sales and tobacco advertising at 40 ‘Smoke Free Hospitals’ in Vietnam

**DOI:** 10.1136/tc-2023-058003

**Published:** 2023-09-05

**Authors:** Joshua David Merritt, Pham Ngoc Yen, Nguyen Thu-Anh, Chau Quy Ngo, Vu Van Giap, Nguyen Viet Nhung, Bui Thi Ha, Ma Thu Thuy, Nguyen Thuy Anh, Nguyen Thuy An, Guy Barrington Marks, Joel Negin, Kavindhran Velen, Greg James Fox

**Affiliations:** 1 Sydney Medical School, Faculty of Medicine and Health, University of Sydney, Sydney, New South Wales, Australia; 2 Respiratory and Environmental Epidemiology Group, Woolcock Institute of Medical Research, Hanoi, Vietnam; 3 Department of Internal Medicine, Hanoi Medical University, Hanoi, Vietnam; 4 Respiratory Center, Bach Mai Hospital, Hanoi, Vietnam; 5 Respiratory Department, Hanoi Medical University, Hanoi, Vietnam; 6 Medical School, Vietnam National University, Hanoi, Vietnam; 7 Vietnam Steering Commitee on Smoking and Health (VINACOSH), Ministry of Health, Hanoi, Vietnam; 8 Respiratory and Environmental Epidemiology Group, Woolcock Institute of Medical Research, Glebe, New South Wales, Australia; 9 South Western Sydney School of Clinical Medicine, University of New South Wales, Sydney, New South Wales, Australia; 10 Sydney School of Public Health, The University of Sydney, Sydney, New South Wales, Australia

**Keywords:** Surveillance and monitoring, Low/Middle income country, Environment, Advertising and Promotion, Prevention

## Abstract

**Background:**

Tobacco remains the leading cause of preventable death globally. Vietnam’s 2012 Law on Prevention and Control of Tobacco Harms establishes all healthcare facilities as smoke-free environments. We aimed to evaluate the implementation of these policies within health facilities across Vietnam.

**Methods:**

A cross-sectional study was undertaken at 40 central, provincial, district and commune healthcare facilities in four provinces of Vietnam. The presence of tobacco sales, smoke-free signage, evidence of recent tobacco use and smoking behaviours by patients and staff were observed over a 1-week period at multiple locations within each facility. Adherence with national regulations was reported using descriptive statistics.

**Results:**

23 out of 40 facilities (57.5%) followed the requirements of the national smoke-free policy regarding tobacco sales, advertising and signage. Smoking was observed within health facility grounds at 26 (65%) facilities during the observation period. Indirect evidence of smoking was observed at 35 (88%) facilities. Sites where smoking was permitted (n=2) were more likely to have observed smoking behaviour (relative risk (RR) 2.16, 95% CI 1.83 to 2.56). Facilities where tobacco was sold (n=7) were more likely to have smoking behaviour observed at any of their sites (RR 1.53, 95% CI 0.93 to 2.51).

**Conclusions:**

Implementation of current smoke-free hospital regulations remains incomplete, with widespread evidence of smoking observed at three levels of the Vietnamese healthcare facilities. Further interventions are required to establish the reputation of Vietnamese healthcare facilities as smoke-free environments.

**WHAT IS ALREADY KNOWN ON THIS TOPIC**

- Tobacco use remains the leading preventable cause of death worldwide.
- WHO Framework Convention of Tobacco Control commits countries, including Vietnam, to establishing healthcare facilities as smoke-free environments.
- There have been studies suggesting a reduction in secondhand smoke exposure in Vietnam since the implementation of the Law on Prevention and Control of Tobacco Harms in 2012, but a paucity of data on the adherence of healthcare facilities to the Establishment of Smoke Free Hospitals Policy.
- There is a lack of global data on relative implementations of smoke-free hospital policies broken down by different levels of hospital size in the healthcare system.

**WHAT THIS STUDY ADDS**

- This multicentre study evaluates the implementation of smoke-free policies in a representative sample of hospitals in four tiers of the Vietnamese healthcare system in both the northern and southern regions of Vietnam.
- This study employed both direct observation and indirect assessment of smoking within facilities, increasing the accuracy of the assessment of recent tobacco use in the facilities.
- This study demonstrated a significant gap between policy and practice in Vietnam, highlighting the need for strengthened policies and enforcement of smoke-free hospital policies.

**HOW THIS STUDY MIGHT AFFECT RESEARCH, PRACTICE OR POLICY**

- This study establishes a number of key factors as possible targets for future intervention research.
- This study establishes a baseline of compliance for Vietnamese healthcare facilities to the national policy and a methodology for future evaluation of other interventional studies.

## Introduction

 Tobacco use remains the leading preventable cause of death worldwide, with 7 million deaths directly attributed to tobacco smoke annually including approximately 890 000 non-smokers exposed to secondhand smoke.^1^ Tobacco use has known health impacts, especially increasing the risk of cardiovascular disease and respiratory disease.^2^,^5^ Tobacco also contributes to infectious disease burden, with a study in Taiwan showing current smokers having twice the odds of developing active tuberculosis compared with non-smokers.^6^

Tobacco control in healthcare facilities is a top public health priority, given the detrimental effect smoking can have on inpatients and the importance for the health system to model healthy behaviour. Interventions to reduce smoking at healthcare facilities have been shown to improve staff education and reducing staff smoking rates.^7^ WHO Framework Convention of Tobacco Control commits countries, including Vietnam, to establishing healthcare facilities as smoke-free environments.^8^

Vietnam is a middle-income country where 45.3% of males were current smokers in 2015.^9^ The annual number of deaths attributable to smoking in Vietnam is expected to exceed 50 000 by 2023.^10^ The Vietnamese government has formally endorsed smoke-free health facilities, including through the 2008 Establishment of Smoke Free Hospitals Act^11^ and the 2012 Law on Prevention and Control of Tobacco Harms.^12^ Decree 176/2013/ND-CP regulates smoke-free areas, including hospitals, and provides sanctions for violating smoke-free areas and tobacco sales. Under sections 23–27 of the decree, smoking and tobacco sales are prohibited in any public healthcare facility and tobacco advertising is prohibited nationally, except at the point of sale.

Smoking rates in the general community have been characterised,^9^ with data suggesting a reduction in exposure to secondhand smoke since 2012.^13 14^ However, there is a paucity of data regarding the adherence of healthcare facilities to national regulations. Evidence is needed to understand whether national smoke-free hospital policies are operating as intended.^15^

This study aimed to evaluate the implementation of national Smoke Free Hospitals policy and smoking behaviours within the grounds of 40 healthcare facilities across four provinces of Vietnam.

## Methods

### Study design and setting

This cross-sectional study was conducted within 40 government health facilities in four of Vietnam’s 63 provinces and municipalities: Hanoi and Thanh Hoa in the North, and Ca Mau and Ho Chi Minh City in the South. These provinces were selected to represent the geographic diversity of Vietnam, comprising one predominantly rural and one urban province each from the North and South. Vietnam’s two largest cities were included. Participating facilities are shown in table 1 and were selected at random in their provinces with a probability in proportion to the population in order to remain representative of those provinces. The total population of the participating provinces was 20 376 800 people, comprising 22% of the country’s population.^16^

**Table 1:** Description of participating health facilities and observation sites*

	All facilities		Central and provincial facilities		District health facilities		Commune health facilities	
	Facilities	Observation sites	Facilities	Observation sites	Facilities	Observation sites	Facilities	Observation sites
	n (%)	n (%)	n (%)	n (%)	n (%)	n (%)	n (%)	n (%)
**Total sites**	40 (100)	163 (100)	8 (100)	43 (100)	16 (100)	72 (100)	16 (100)	48 (100)
Region								
North	20 (50)	80 (49.1)	4 (50)	23 (53.5)	8 (50)	33 (45.8)	8 (50)	24 (50)
South	20 (50)	83 (50.9)	4 (50)	20 (46.5)	8 (50)	39 (54.2)	8 (50)	24 (50)
Setting								
Urban	21 (52.5)	89 (54.6)	7 (87.5)	38 (88.4)	7 (43.8)	30 (41.7)	7 (43.8)	21 (43.8)
Rural	19 (47.5)	74 (45.4)	1 (12.5)	5 (11.6)	9 (56.3)	42 (58.3)	9 (56.3)	27 (56.3)

* Facilities defined as individual healthcare centres; observation sites defined as locations in those centres where observations were recorded.

### Study sites

The public healthcare system in Vietnam consists of four levels of facilities: central, provincial, district and commune facilities. In each participating province, all central (national level) and provincial hospitals were included. District health facilities were selected from among all districts in each province using random sampling. Within selected districts, two commune health posts were also selected at random.

Within each health facility, observation sites were selected following a discussion with a senior clinician at the facility. Sites where staff were aware smoking might take place were selected, including outdoor courtyards, gardens, staff or public cafeterias, hospital entrances, patient wards, outdoor walkways, meeting rooms, parking areas and corridors. At each facility, three to seven sites were selected, depending on the availability of physically separate locations meeting these criteria. These are shown in table 1.

### Data collection

Four aspects of the Smoke Free Hospitals Policy were evaluated.

#### Observation of adherence to smoke-free signage

At each selected site, data were collected about the type of site and whether there were any visible smoke-free signs, of any size and in any location, present in the predetermined area. Although national legislation required health facilities to be smoke free, some sites had been informally deemed permissible for smoking by the hospital director. Data were collected about each site to note whether this was the case.

#### Identification of tobacco advertising or sites of tobacco sales within the facility

Data were recorded about whether tobacco advertising was visible, and whether there were any methods available for the purchase of tobacco at the site, such as within a hospital canteen.

#### Direct observation of smoking behaviour

Each site was observed by study staff independent of the healthcare facility on five consecutive weekdays, with the first observation period not on a Monday (to ensure a weekend was captured). Observation times were randomly generated, including at least one observation period coinciding with staff lunch breaks. Each site was observed for a period of 25 min at a time, and was observed twice each day for those five consecutive days.

Healthcare staff were not informed that observation was taking place, nor the time at which observations would occur. The trained observer was not known to health facility staff, and positioned themselves in a non-obtrusive position with line of sight to the entire site.

During each observation period, the observer counted the number of people entering the site, and whether each person had any features indicating they were a staff member (such as a white coat or hospital badge). The number of people smoking was directly observed by research staff, although the specific form of tobacco used not reported.

#### Indirect evidence of recent smoking

Prior to the first observation period, the observer collected all cigarette butts that had been discarded in the designated areas. During each subsequent observation period, the observer collected and counted all cigarette butts, including partial butts, that had been newly deposited in the designated area, including those in bins or ashtrays. This approach technique identified evidence of smoking between observation periods.

### Quality assurance

During two observation periods, a second senior researcher simultaneously counted smoking behaviours to ensure the accuracy of the observations, and ensure standardisation of the observation methods. If the total number of observations made by the senior researcher did not completely agree with those of the primary observer, the senior researcher’s results were used. If needed, training was continued until there was 100% agreement between both observers. A total of five staff collected data across all of the facilities.

### Data analysis

Simple descriptive statistics were calculated to determine the number of sites with smoke-free signage. The number of sites that allowed the sale of tobacco and sites that contained any tobacco product advertising was calculated. A facility was designated as a smoke-free facility if it had all of the following: no tobacco advertisements, no evidence of smoking and no evidence of tobacco sales. Outcomes at central and provincial-level health facilities were combined, given the small number of central health facilities included in the study.

Descriptive statistics were used to evaluate the number of observation periods where one or more people were observed to be smoking, and the number of sites where any staff was observed smoking.

The number of sites where at least one cigarette butt was found during the entire observation period was calculated.

The effect of smoke-free policies, smoke-free signage, tobacco sales and staff smoking on direct and indirect measures of smoking at sites was calculated using an unadjusted relative risk (RR). Analyses were performed using SPSS (V.25, IBM).

## Results

Site visits were conducted between March 2018 and October 2018 at 163 observation sites across 40 healthcare facilities. Overall, 105 out of 163 (64.4%) observation sites within the 40 health facilities had smoke-free signage visible (table 2). Six of the 40 facilities (15%) had all selected sites with some form of smoke-free signage. Two health facilities had observation sites where tobacco use had been deemed permissible by local authority, and there were three facilities with observation sites where it was not known whether smoking was permissible or not. There were 35 out of 40 facilities (87.5%) with a smoke-free policy that applied to all observation sites. In five facilities, staff were informally permitted to smoke in a designated location on hospital grounds by the hospital director, despite the applicable legislation.

**Table 2:** Observations of tobacco sales and advertising at healthcare facilities

	Total facilities	Central or provincial facilities	District facilities	Commune facilities
**Total facilities**	**n=40**	**n=8**	**n=16**	**n=16**
Cigarettes sold				
Yes, n (%)	7 (17.5)	1 (12.5)	6 (37.5)	0 (0)
No, n (%)	33 (82.5)	7 (87.5)	10 (62.5)	16 (100)
Tobacco advertising present				
Yes, n (%)	11 (27.5)	5 (62.5)	5 (31.3)	1 (6.3)
No, n (%)	28 (70.0)	3 (27.5)	10 (62.5)	15 (93.8)
Missing, n (%)	1 (2.5)*	0 (0)	1 (6.3)*	0 (0)
**Within observation sites** **†**	**n=163**	**n=43**	**n=72**	**n=48**
Tobacco use permitted by local authority				
No, n (%)	158 (96.9)	43 (100)	68 (94.4)	47 (97.9)
Yes, n (%)	2 (1.2)	0 (0)	1 (1.4)	1 (2.1)
Don’t know, n (%)	3 (1.8)	0 (0)	3 (4.2)	0 (0)
Smoke-free signs visible				
Yes, n (%)	105 (64.4)	36 (83.7)	42 (58.3)	27 (56.3)
No, n (%)	56 (34.4)	7 (16.3)	29 (40.3)	20 (41.7)
n/a‡, n (%)	2 (1.2)	0 (0)	1 (1.4)	1 (2.1)

* In one facility, the advertising status was missing for one observation site, but advertising was not identified at other sites in this facility.

† A breakdown of observation sites by facility is available in table 1.

‡ Smoking was informally permitted at these sites.

Some form of tobacco advertising was present at 17 observation sites across 11 facilities. Overall, 28 out of the 40 included facilities (70%) had no evidence of tobacco advertising. Seven observation sites across seven (18%) different facilities allowed sale of tobacco in some form. Overall, 23 facilities (57.5%) followed regulations regarding tobacco sales, advertising and signage (table 2). Data were analysed at a facility level due to the likely correlation between observation sites within the same facility.

### Direct observation of smoking behaviour

Out of 819 observation periods (341 hours of observation), staff were observed smoking 17 times (2.1% of observations) across nine facilities (22.5%). Across the 163 observation sites, 83 (52.1%) had no periods where smoking was observed. Overall, 14 facilities (35.0%) had no observation sites where a person was observed smoking (table 3).

**Table 3:** Direct observation of smoking behaviour within health facilities

	Total	Central/provincial	District	Commune
**Smoking behaviour by observation periods**				
Observation periods (n)	819	235	344	240
Staff smoking observed (total)				
Observation periods where staff were observed smoking, n (%)	17 (2.1)	5 (2.1)	12 (3.5)	0 (0)
Observation periods where no staff were observed smoking, n (%)	802 (97.9)	230 (97.9)	332 (96.5)	240 (100)
Smoking observed (all hospital attendees)				
Observation periods where smoking was observed, n (%)	233 (28.4)	112 (47.7)	113 (32.8)	8 (3.3)
Observation periods where no smoking was observed, n (%)	586 (71.6)	123 (52.3)	231 (67.2)	232 (96.7)
Observation sites*	**n=163**	**n=43**	**n=72**	**n=48**
Staff smoking observed				
Observation sites where staff were observed smoking in at least one observation period, n (%)	9 (5.5)	3 (7.0)	6 (8.3)	0 (0)
Observation sites where no staff were observed smoking in any observation period, n (%)	154 (94.5)	40 (93.0)	66 (91.7)	48 (100)
Total smoking observed				
Observation sites where smoking was observed in at least one observation period, n (%)	78 (47.9)	30 (69.8)	42 (58.3)	6 (12.5)
Observation sites where no smoking was observed in any observation period, n (%)	85 (52.1)	13 (30.2)	30 (41.7)	42 (87.5)
**Facilities†**	**n=40**	**n=8**	**n=16**	**n=16**
Staff smoking observed				
Facilities where staff were observed smoking at any observational site during at least one observation period, n (%)	9 (22.5)	3 (37.5)	6 (37.5)	0 (0)
Facilities where no staff were observed smoking at any observation site during any observation period, n (%)	31 (77.5)	5 (62.5)	10 (62.5)	16 (100)
Total smoking observed				
Facilities where smoking was observed at any observational site during at least one observation period, n (%)	26 (65.0)	8 (100)	15 (93.8)	3 (18.8)
Facilities where no smoking was observed at any observation site during any observation period, n (%)	14 (35.0)	0 (0)	1 (6.3)	13 (81.3)

* Sites are said to have observed smoking if at least one observation period at that site had observed smoking.

† Facilities are said to have observed smoking if at least one observation site at that facility had observed smoking.

### Indirect evidence of smoking

Cigarette butts were collected from 92 observation sites (56.4%) (table 4). Only five out of 40 facilities (12.5%) had no cigarette butts collected at any observation period.

**Table 4:** Indirect measures of smoking behaviour by observation periods, observation sites and facilities

	Total	Central/provincial	District	Commune
**Within observation periods**	**n=819**	**n=235**	**n=344**	**n=240**
Cigarette butts collected				
One or more found during an observation period, n (%)	352 (43.0)	140 (59.6)	187 (54.4)	25 (10.4)
None found during an observation period, n (%)	467 (57.0)	95 (40.4)	157 (45.6)	215 (89.6)
At observation sites*	**n=163**	**n=43**	**n=72**	**n=48**
Cigarette butts collected				
One or more found at a site during any observation period, n (%)	92 (56.4)	31 (72.1)	46 (63.9)	15 (31.3)
None found at a site during any observation period, n (%)	71 (43.6)	12 (27.9)	26 (36.1)	33 (68.8)
**At healthcare facilities†**	**n=40**	**n=8**	**n=16**	**n=16**
Cigarette butts collected				
One or more found at any observation site in a facility during any observation period, n (%)	35 (87.5)	8 (100)	16 (100)	11 (68.8)
None found at any observation site in a facility during any observation period, n (%)	5 (12.5)	0 (0)	0 (0)	5 (31.3)

* Sites are said to have cigarette butts found if at least one observation period at that site had cigarette butts found.

† Facilities are said to have cigarette butts found if at least one observation site at that facility had cigarette butts found.

### Comparative analyses

Table 5 shows the predictors of directly and indirectly observed smoking behaviours at participating sites. Smoking was more likely to be observed at sites where tobacco use was permitted by local authority. These sites had an RR of 2.16 (95% CI 1.83 to 2.56) of observed smoking behaviour in at least one observation period compared with sites where smoking was not permitted. Sites where tobacco use was permitted had an RR of 1.82 (95% CI 1.58 to 2.10) of having cigarette butts found on at least one occasion compared with sites where smoking was not permitted.

**Table 5:** Univariable analysis of factors associated with direct and indirect measures of smoking behaviour at observation sites

Factors	At least one smoker observed	No smokers observed	Relative risk of at least one smoker being observed		At least one cigarette butt collected	No cigarette butts collected	Relative risk of at least one cigarette butt being collected	
	n (%)	n (%)	RR	95% CI	n (%)	n (%)	RR	95% CI
Smoking permitted by local authority								
No	73 (46.2)	85 (53.8)	Ref		87 (55.1)	71 (44.9)	Ref	
Yes	5 (100)	0 (0)	**2.16**	**1.83 to 2.56**	5 (100)	0 (0)	**1.82**	**1.58 to 2.10**
Visible smoke-free signage								
No	33 (56.9)	25 (43.1)	Ref		44 (75.9)	14 (24.1)	Ref	
Yes	45 (42.9)	60 (57.1)	0.75	0.55 to 1.03	48 (45.7)	57 (54.3)	0.60	0.47 to 0.78
Tobacco sold at this site								
No	73 (46.8)	83 (53.2)	Ref		86 (55.1)	70 (44.9)	Ref	
Yes	5 (71.4)	2 (28.6)	1.53	0.93 to 2.51	6 (85.7)	1 (14.3)	**1.56**	**1.11 to 2.17**
Tobacco sold anywhere in facility								
No	61 (46.2)	71 (53.8)	Ref		73 (55.3)	59 (44.7)	Ref	
Yes	17 (54.8)	14 (45.2)	1.19	0.78 to 1.81	19 (61.3)	12 (38.7)	1.16	0.71 to 1.87
Tobacco advertised at this site								
No	69 (47.9)	75 (52.1)	Ref		81 (56.3)	63 (43.8)	Ref	
Yes	9 (47.4)	10 (52.6)	0.99	0.60 to 1.64	11 (57.9)	8 (42.1)	1.03	0.68 to 1.55
Staff observed smoking at this site								
No*	44.8 (69)	85 (55.2)	Ref		84 (54.5)	70 (45.5)		
Yes*	9 (100)	0 (0)	2.23*	1.87 to 2.66*	8 (88.9)	1 (11.1)	**1.63**	**1.24 to 2.14**

* Excludes staff observed smoking.

† Numbers in bold indicate p<0.05.

RR, relative risk.

Sites with visible smoke-free signage were less likely to have observed smoking behaviour compared with sites with no smoke-free signs (RR 0.75, 95% CI 0.55 to 1.03). Indirect evidence of smoking (defined as having at least one cigarette collected) was less likely at sites with smoke-free signs (RR 0.60, 95% CI 0.47 to 0.78).

Tobacco advertising had little impact on either direct or indirect observations of smoking behaviour. There was no difference in observed smoking (RR 0.99, 95% CI 0.60 to 1.64) or indirect smoking observation (RR 1.03, 95% CI 0.68 to 1.55) between sites where tobacco advertising was present compared with those without.

Smoking behaviour was more likely to be observed at sites where tobacco products were available for purchase (RR 1.53, 95% CI 0.93 to 2.51) compared with sites where they were not available. Indirect evidence smoking was more likely to be present at sites where tobacco was sold (RR 1.56, 95% CI 1.11 to 2.17).

Facilities at which tobacco was sold were more likely to have indirect evidence of smoking at least one site, compared with those where tobacco was not sold at any of the chosen sites (RR 1.16, 95% CI 0.71 to 1.87). Smoking behaviour was more likely to be observed at facilities where tobacco was sold (RR 1.19, 95% CI 0.78 to 1.81).

Patients and visitors were more likely to be observed smoking at sites where a staff member smoked (RR 2.23, 95% CI 1.87 to 2.66). Indirect evidence of smoking was also more likely where a staff member was observed smoking (RR 1.63, 95% CI 1.24 to 2.14).

## Discussion

This cross-sectional study evaluated smoking behaviours, signage and tobacco sales in 40 healthcare facilities in northern and southern Vietnam. A substantial proportion of healthcare facilities demonstrated evidence of smoking behaviour on-site, tobacco sales and tobacco advertising despite national prohibitions. Sites where healthcare workers smoked were more likely to also have smoking by other visitors to the hospital.

These findings are consistent with previous studies in Vietnam which have found implementation of smoke-free policies has limited reach. In Hanoi, it was found that 75.8% of customers did not see, or rarely saw, no-smoking signs in restaurants.^17^ Further, 17.7% of restaurant patrons frequently observed direct tobacco advertising in restaurants and almost two-thirds were sometimes or always exposed to secondhand smoke when visiting a restaurant. Nevertheless, in workplaces secondhand smoke exposure dropped from 55.9% in 2010 to 42.6% in 2015.^13^ Study findings are also consistent with research performed in other countries. In the UK, only one in 16 hospitals were fully compliant with national guidelines,^18^ and just 38% of health facilities in Indonesia were compliant with six criteria stipulated by national policies.^19^

Studies in other settings have also demonstrated limited compliance with smoke-free policies.^20^ In Spain, 64% of patients were aware of a smoke-free hospitals policy.^21^ The same showed a reduction in smoking prevalence following the implementation of smoke-free hospitals. An Australian survey found limited enforcement of smoke-free policies by healthcare workers.^22^ Important aspects of policies to strengthen smoke-free hospitals include clear communication of the policy, clear signposting, surveillance of compliance and a coercivity policy for circumstances where breaches occur.

This study has important policy implications. Given the gaps between policy and practice, further interventions are required to improve compliance with current regulations. Structured health system interventions have led to substantial improvements in the enforcement of smoke-free hospital regulations in Australia.^23^ Similar approaches may be effective in Vietnam. Formal evaluation of hospitals’ compliance with national policies may also be beneficial and is a component of WHO MPOWER framework, the 6 main measures of tobacco intervention set forth by the WHO Framework Convention on Tobacco Control.^8^ Frequent external oversight of health facility compliance with these policies, with feedback to the health facility leaders, may contribute to greater degrees of compliance. Additionally, greater investment is required within the health system to strengthen enforcement and enhance promotion of smoke-free policies at all levels of the health system.

Our finding that smoking was more common in sites where staff smoke highlights the importance of reducing staff smoking rates as a method of reducing overall smoking within these health facilities. Further investment in supporting smoke-free hospitals and encouraging staff to stop smoking will be an important priority to support tobacco control in Vietnamese hospitals.^24^ Current Vietnamese law^12^ designates local police as the authorities able to issue fines for violation of smoking behaviour. In practice, there is limited capacity for police to undertake this work. As a result, during this study there was no response to violations of smoking policy. Therefore, our study indicates that further efforts to strengthen the regulation and increase the capacity for local enforcement in each hospital will be required to reduce smoking in health facilities.

This study has a number of strengths. First, the findings are broadly representative of the Vietnamese healthcare system, with data taken from all four levels of facilities (national, provincial, district and commune). This study also demonstrated the feasibility of a standardised approach evaluating smoke-free hospitals, adapted to the Vietnamese healthcare system. The approach may be reproduced in the future to evaluate the effect of changing tobacco control regulations.

The study has several limitations. First, some health facility staff may have been aware the study was being conducted, since the local facility director had provided prior approval to conduct the study. To mitigate this risk, observation times were generated randomly throughout the week. Observers were trained to be discreet and wearing plain clothes, and were not known to local staff. Nevertheless, a Hawthorne effect was possible. A second limitation is the method of site selection. Sites were chosen based on the assessment of research staff regarding where people may choose to smoke. Some common smoking sites may have been missed, potentially underestimating the true smoking prevalence. We were also not able to collect data regarding the types of tobacco product used. Only cigarette butts were included in indirect measurements, which may underestimate smoking among users of traditional water pipes, comprising around 25% of smoking in Vietnam.^25^

Further research is required to evaluate the effectiveness of comprehensive smoke-free policies, including smoking cessation interventions for healthcare workers and patients.

In conclusion, our study demonstrated a gap between policy and practice regarding the implementation of smoke-free hospitals. Given the importance of healthcare facilities to normalising smoke-free environments more broadly, these interventions will have important benefits beyond the healthcare facilities. A renewed focus on smoke-free hospitals will help reduce the growing harm due to tobacco in Vietnam and reverse the long-term mortality due to tobacco.

## Data Availability

Data are available upon reasonable request.
